# Cross-sectional study of antimicrobial use and treatment decision for preweaning Canadian dairy calves

**DOI:** 10.3168/jdsc.2021-0161

**Published:** 2021-11-25

**Authors:** Tamaki Uyama, David F. Kelton, Emma I. Morrison, Ellen de Jong, Kayley D. McCubbin, Herman W. Barkema, Simon Dufour, Javier Sanchez, Luke C. Heider, Stephen J. LeBlanc, Charlotte B. Winder, J.T. McClure, David L. Renaud

**Affiliations:** 1 Department of Population Medicine, University of Guelph, Guelph, ON, N1G 2W1, Canada; 2 Department of Production Animal Health, Faculty of Veterinary Medicine, University of Calgary, Calgary, AB, T2N 4N1, Canada; 3 Faculté de médecine vétérinaire, Université de Montréal, St-Hyacinthe, QC, J2S 2M2, Canada; 4 Department of Health Management, Atlantic Veterinary College, University of Prince Edward Island, Charlottetown, PE, C1A 4P3, Canada

## Abstract

Antimicrobials should be used prudently in farm animals to prevent the development of resistant bacteria in both humans and animals. The objective of this study was to investigate Canadian dairy producers' practices for antimicrobial use in the treatment of disease in preweaning dairy calves. In-person questionnaires were administered to 144 dairy producers across 5 provinces in Canada between July 2019 and August 2020. Almost all (96%) producers used antimicrobials to treat calves with respiratory disease, but only 27% indicated they had a written treatment protocol for respiratory disease. Most (95%) of these protocols for respiratory disease were developed with input from the herd veterinarian. Seventy-four percent of producers used antimicrobials to treat calf diarrhea, with 37% of producers having a written treatment protocol for calf diarrhea with input from the herd veterinarian. The combinations of signs adopted by the producers for antimicrobial treatment in calf respiratory disease and diarrhea were evaluated based on findings from other studies. More than half (56%) of producers who used antimicrobials for calf respiratory disease decided to use antimicrobials by evaluating multiple clinical signs. Eighty-two percent of producers who used antimicrobials for calf diarrhea made decisions based on systemic signs of disease, presence of bloody stool, no response to previous treatment, or on the recommendation from the herd veterinarian. Producers with a written treatment protocol had 3 to 7 times greater odds of using antimicrobials based on multiple signs or systemic signs of disease compared with those without a protocol. Further research may investigate other calf management practices related to decision-making by producers in using antimicrobials to improve antimicrobial stewardship on dairy farms.

## Highlights

• More than half of producers used multiple signs when treating calf pneumonia.
• More than half of producers included systemic signs when treating calf diarrhea.
• Farmers with a treatment protocol used multiple signs for antimicrobial use (bovine respiratory disease).
• Farmers with a treatment protocol used systemic signs for antimicrobial use (scours).

## Graphical Abstract

**Figure:**
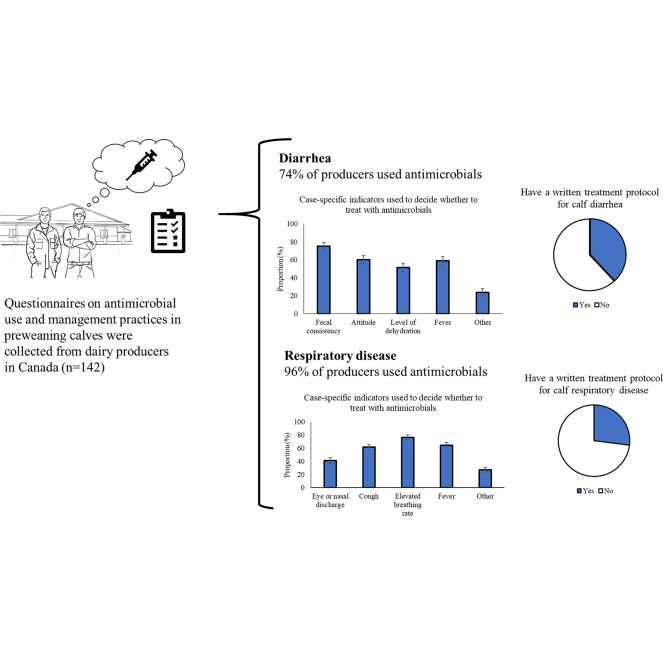
**Summary:** Antimicrobials are commonly used to treat dairy calves, which increases the risk of development of antimicrobial resistance and, as a result, shedding resistant bacteria. However, it is unclear how dairy producers decide to use antimicrobials for treating their calves. Questionnaires were administered to 144 dairy producers across 5 provinces in Canada. Nearly all producers used antimicrobials to treat calf respiratory disease and almost three-fourths of producers used antimicrobials to treat calf diarrhea. Less than 40% of dairy producers had written treatment protocols for calf diseases. Improvements can be made for more prudent antimicrobial use in Canadian dairy calves.

It is important to understand antimicrobial use (**AMU**) in food animals because of its role in the development of antimicrobial resistance (**AMR**), which can have a significant impact on human and animal health (Tang et al., 2017). In the dairy industry, most AMU is for treatment of mastitis during lactation and at dry-off (Nobrega et al., 2017; Redding et al., 2019; Lardé et al., 2021). However, AMU in calves is also a potentially important contributor to overall farm AMU because diarrhea and respiratory disease are common diseases in calves treated with antimicrobials (USDA-APHIS, 2018; Lardé et al., 2021). In one study, dairy veterinarians perceived there is unnecessary use of antimicrobials in preweaning calves on commercial US dairy farms (Webb et al., 2019). Moreover, few studies have focused on AMU in dairy calves, including the criteria used by producers in making treatment decisions.

Various calf health scoring systems have been developed, such as the respiratory scoring systems developed by McGuirk and Peek (2014) and Love et al. (2014) for preweaning calves and that of Maier et al. (2019) for weaned calves, to standardize calf disease diagnosis. Based on these clinical scoring systems, decision trees have been developed to guide rational, selective treatment with antimicrobials. For respiratory disease, Love et al. (2014) allocated differing weights to each of following symptoms: presence of cough (2 points), nasal discharge (4 points), eye discharge (2 points), fever (2 points), abnormal respiration (2 points), or ear and head carriage (5 points). Using this system, a case of respiratory disease was defined as ≥5 points, which correctly classified 90% of calves (Love et al., 2014). Maier et al. (2019) suggested another scoring system, where cough (2 points), abnormal respiration (1 point), low body condition (5 points), sunken eyes (4 points), and a 24-h ambient temperature range of more than 15°C (1 point) were used, with the cut-off at 2 points for a case of respiratory disease. With respect to diarrhea, Berge et al. (2009) and Gomez et al. (2017) highlighted that the presence of fever, bloody stool, or depressed attitude, and not solely fecal consistency, should be used to determine when to use antimicrobials. When these decision trees were applied on commercial farms, they reduced overall AMU without adversely affecting calf mortality or the number of days with diarrhea (Berge et al., 2009; Gomez et al., 2017). Despite the availability of these tools, there are little data to indicate the extent to which they are used on commercial dairy farms.

The primary objective of this study was to describe decision-making by Canadian dairy producers regarding AMU for treatment of diarrhea and respiratory disease in preweaning dairy calves. A secondary objective was to compare herd characteristics of producers who used different case-specific information for treatment decisions for calves with respiratory disease and diarrhea. We hypothesized that farms with a treatment protocol for calf disease used multiple clinical signs to make decisions on antimicrobial treatment compared with those without a protocol.

This cross-sectional study was conducted on Canadian dairy farms between July 2019 and August 2020. The study received approval from the University of Guelph Research Ethics Board (#19-04-005) and is reported using the STROBE-Vet guideline (O'Connor et al., 2016). A total of 144 commercial dairy farms were recruited through convenience sampling across 5 provinces in Canada (British Columbia, Alberta, Ontario, Québec, and Nova Scotia). Farms enrolled in the study were originally recruited for a surveillance research project by the Canadian Dairy Network for Antimicrobial Stewardship and Resistance (CaDNetASR), which is a 5-year project that began in July 2019 to investigate AMU and AMR on Canadian dairy farms. The sample size was determined based on the larger project on AMU, where the number of farms designated to each sentinel site of the FoodNet Canada program was considered. Producers were eligible to participate in the current study if they raised their own replacement heifers, were willing to provide their antimicrobial purchasing information, and were enrolled in DHI milk recording through Lactanet (Guelph, ON, Canada).

Producers completed 4 questionnaires during the first 2 farm visits that were conducted within 2 wk of each other. Questionnaires were developed by the CaDNetASR group and administered to producers by research staff during an in-person visit. For this study, only questions related to AMU for treating calf diseases, presence of written treatment protocols for calf diseases, herd demographics, and housing facilities for preweaning calves were used and are shown here: https://doi.org/10.5683/SP2/RYDAHV. All responses were entered in an app version of the REDCap system (Vanderbilt University, Nashville, TN) on an iPad Air (Apple Inc.) or a paper copy of the questionnaire and later uploaded to the CaDNetASR REDCap system. All relevant responses were exported to an Excel (Microsoft Corp.) spreadsheet for further analysis and validated for any missing data.

Statistical analyses were completed using Stata 16 (StataCorp LLC). Descriptive statistics included proportions for categorized variables and median and interquartile range (**IQR**) for continuous variables. Described variables were herd size, preweaning calf housing, having written treatment protocols for calf diseases and whether they were developed with input from the herd veterinarian, AMU for calf diseases, and case-specific indicators used for treating calf diarrhea and respiratory disease with antimicrobials. Herd size was the number of milking cows on the farm on the day of visit. After scrutinizing responses for housing type for preweaning calves, responses were categorized into individual indoors, individual outdoors, group housing either indoors or outdoors, and a combination of multiple housing types. Tethered calves were considered to be calves housed individually indoors.

Combinations of case-specific indicators selected for calf diseases were further explored for calf respiratory disease and diarrhea. With respect to calf respiratory disease, the responses by producers were dichotomized as using “≥3 signs” or “<3 signs,” based on the scoring system by Love et al. (2014). With respect to calf diarrhea, responses by producers were dichotomized as using “systemic sign” or “no systemic sign” where the former group used at least one of the signs (fever, attitude, bloody stool, recommendation from veterinarian, no response to previous treatments) and the remaining responses were included in the latter group based on the criteria used by other studies (Berge et al., 2009; Gomez et al., 2017; McDougall et al., 2017).

Two multivariable logistic regression models were built using the following dichotomous dependent variables: (1) “≥3 signs” or “<3 signs” used for AMU in calf respiratory disease; and (2) “systemic sign” or “no systemic sign” used for AMU in calf diarrhea. Only responses that used antimicrobials for each disease were included in the analysis. Explanatory variables were herd size, presence of a written treatment protocol for each disease, veterinary input on written treatment protocol, and calf housing type. Linearity between herd size as a continuous variable and the log odds of the dependent variables was assessed by plotting a locally weighted smoothing curve. When the assumption of linearity was violated, herd size was categorized into 3 groups (small, medium, large) based on mean herd size in western and eastern Canadian dairy herds from national statistics (Progressive Dairy, 2019). Small farms (≤82 cows) were defined as farms with a herd size smaller than the eastern average, resulting in 50 farms. Large farms (≥164 cows) were defined as farms with a herd size larger than the western average, resulting in 37 farms. Medium-sized farms were those that milked between 83 and 163 cows, resulting in 55 farms. Pearson chi-squared test was used to assess all explanatory variables for collinearity, with variables having a significant relationship (*P* < 0.05) being considered collinear. A written treatment protocol and having herd veterinarian input on the protocol were collinear for each disease; therefore, the latter variable was omitted from the analysis.

Univariable analysis was conducted between the dependent variable and each explanatory variable using a logistic regression model, and those explanatory variables associated with *P* < 0.20 were included in the final model. Manual backward selection was performed and variables with *P* < 0.05 were retained in the final model. Interaction terms were evaluated between herd size and presence of a written protocol because we expected that larger farms would be more likely to have a written treatment protocol; a significant interaction term was not found in any of the models. Confounding was assessed and a variable was retained in the final model if its removal changed the coefficients by >20%. A Pearson goodness-of-fit test was used to assess the fit of the model with *P* < 0.05; all models fit the data.

A total of 142 of the 144 farms completed responses to the questions of interest and were included in the analysis. Two responses were omitted from the analysis due to missing data. Farms were located in British Columbia (n = 28), Alberta (n = 30), Ontario (n = 31), Québec (n = 29), and Nova Scotia (n = 24). Median herd size was 107 milking cows (IQR: 68–175; range: 36–560). Among included study farms, 48 (34%) housed preweaning calves individually indoors, 22 (15%) housed calves individually outdoors, 22 (15%) housed calves in groups either indoors or outdoors, and 50 (35%) used a combination of multiple housing types. Thirty-six producers (25%) indicated they had written treatment protocols for both calf diarrhea and respiratory disease, 3 (2%) had a protocol for calf respiratory disease only, 19 (13%) had a written treatment protocol for calf diarrhea only, and the remaining 84 farms (59%) did not have a protocol for either disease.

In total, 136 (96%) farms used antimicrobials to treat calf respiratory disease. Thirty-nine (27%) producers answered they had a written treatment protocol for calf respiratory disease, of which 37 (95%) indicated herd veterinarian input in protocol development. Of the farms that used antimicrobials when treating respiratory disease, 56 (41%) used nasal or eye discharge, 84 (62%) used cough, 104 (76%) used elevated breathing rate, 88 (65%) used fever, and 37 (27%) used other indicators to select cases for antimicrobial treatment. The other indicators (n = 37), which could have included multiple signs in one response, were attitude of the calf (n = 14; 38%), loss of appetite (n = 13; 35%), group treatment (n = 8; 22%), veterinary recommendation (n = 4; 11%), rumination (n = 1; 2.7%), value of the calf (n = 1; 2.7%), and the presence of comorbidities (n = 1; 2.7%). Twenty-three (17%) producers used all 4 respiratory-specific indicators (i.e., nasal or eye discharge, cough, breathing rate, and fever), 15 (11%) used a combination of elevated breathing rate and fever, 13 (10%) used cough, elevated breathing rate, and fever, and 10 (7%) used other signs or a combination of cough and elevated breathing rate. Based on our classification, 76 (56%) used at least 3 signs to make decisions on AMU for calf respiratory disease.

Using at least 3 signs on AMU in respiratory disease had a univariable association with herd size and presence of a written treatment protocol and both were offered to a multivariable model (Table 1). Herd size was not significant and not identified as confounder. Therefore, the variable was removed from the model and no multivariable model was developed. Univariable association showed farms that had a written treatment protocol for calf respiratory disease had higher odds (odds ratio 3.1; 95% CI: 1.4–7.0; *P* = 0.007) of using at least 3 signs when making decisions to use antimicrobials than those without a protocol.

**Table 1:** Univariable association on using at least 3 clinical signs for antimicrobial use in calf respiratory disease (n = 136)

Variable	Farms using â‰¥3 signs, n	Farms using <3 signs, n	Odds ratio	95% CI	*P*-value
Herd size					
Small (36–82 milking cows)	29	17	Referent		
Medium (83–163 milking cows)	26	27	0.56	0.25–1.26	0.16
Large (164–560 milking cows)	21	16	0.77	0.32–1.86	0.56
Presence of treatment protocol for calf respiratory disease					
No	47	50	Referent		
Yes	29	10	3.09	1.36–7.02	0.007
Calf housing type					
Individual indoors	28	19	Referent		
Individual outdoors	11	8	0.93	0.32–2.75	0.90
Group housing	10	11	0.62	0.22–1.74	0.36
Combination	27	22	0.83	0.37–1.87	0.66

In total, 105 (74%) farms indicated they used antimicrobials for the treatment of calf diarrhea, and 55 (39%) of 142 farms had a written treatment protocol for the disease. Among those with a written treatment protocol, 53 (96%) indicated they had input from their herd veterinarian in protocol development. Among farms that used antimicrobials for calf diarrhea, 79 (75%) considered fecal consistency, 63 (60%) attitude, 54 (51%) level of dehydration, 62 (59%) fever, and 25 (24%) used other indicators to select cases for antimicrobial treatment. The other indicators (n = 25), which included multiple signs in one response, were loss of appetite (n = 7; 28%), no response to previous treatments (n = 5; 20%), bloody stool (n = 3; 12%), not ruminating (n = 3; 12%), persistent diarrhea (n = 2; 8%), age (n = 2; 8%), presence of comorbidities (n = 2; 8%), group treatment (n = 1; 4%), decrease in drinking speed (n = 1; 4%), veterinary recommendation (n = 1; 4%), and having cold ears (n = 1; 4%). Nineteen (18%) producers used all 4 categories of diarrhea-specific indicators (i.e., fecal consistency, attitude, level of dehydration, and fever), 12 (11%) used only fecal consistency, 8 (8%) producers used a combination of fecal consistency, attitude, and fever or a combination of fecal consistency and attitude, and 7 (7%) used all 4 indicators along with other signs. Based on our classification, 86 (82%) used systemic signs when making decisions on AMU in calf diarrhea.

There were univariable associations between using systemic signs in AMU for calf diarrhea and herd size, presence of a written treatment protocol for calf diarrhea, and housing type (Table 2). All explanatory variables were put into a multivariable model. Housing type was removed because it was not a confounder and was not significant in the model. Hence, herd size and presence of a written treatment protocol were included in the final multivariable model (Table 3). Medium-sized farms had lower odds (odds ratio: 0.2; 95% CI: 0.05–0.67; *P* = 0.01) of using a systemic sign for making decisions on AMU for calf diarrhea compared with smaller farms. In addition, farms with a written treatment protocol for calf diarrhea had higher odds (odds ratio: 6.8; 95% CI: 1.7–26.8; *P* = 0.006) of using systemic sign on AMU for calf diarrhea than those without a protocol.

**Table 2:** Univariable association on using at least one of the clinical systemic signs (attitude, fever), bloody stool, recommendation by the herd veterinarian, or no response to previous treatments for antimicrobial use in calf diarrhea (n = 105)

Variable	Farms using systemic sign, n	Farms using no systemic sign, n	Odds ratio	95% CI	*P*-value
Herd size					
Small (36–82 milking cows)	36	4	Referent		
Medium (83–163 milking cows)	27	11	0.27	0.08–0.95	0.04
Large (164–560 milking cows)	23	4	0.64	0.15–2.81	0.55
Presence of treatment protocol for calf diarrhea					
No	45	16	Referent		
Yes	41	3	4.86	1.32–17.90	0.02
Calf housing type					
Individual indoors	25	10	Referent		
Individual outdoors	16	2	3.2	0.62–16.54	0.17
Group housing	16	0	—	—	—
Combination	29	7	1.66	0.55–5.00	0.37

**Table 3:** Final multivariable logistic regression on using at least one of the clinical systemic signs (attitude, fever), bloody stool, recommendation by the herd veterinarian, or no response to previous treatments for antimicrobial use in calf diarrhea (n = 105)

Variable	Farms using systemic sign, n	Farms using no systemic sign, n	Odds ratio	95% CI	*P*-value
Herd size					
Small (36–82 milking cows)	36	4	Referent		
Medium (83–163 milking cows)	27	11	0.18	0.05–0.67	0.01
Large (164–560 milking cows)	23	4	0.48	0.10–2.21	0.34
Presence of treatment protocol for calf diarrhea					
No	45	16	Referent		
Yes	41	3	6.83	1.74–26.80	0.006

In this study, almost all dairy producers (96%) used antimicrobials for treatment of calf respiratory disease, and 74% used antimicrobials for treating calf diarrhea. These findings are similar to reports from US dairy herds where 95% of calves with respiratory disease were treated with antimicrobials and 76% of preweaning calves with diarrhea were treated with antimicrobials (USDA-APHIS, 2018). It is important to note, however, that we did not collect data on the frequency of AMU in calf diseases (e.g., always or sometimes) and this remains a knowledge gap for Canadian farms. Notably, less than half of producers indicated they had a written treatment protocol for calf diseases. Other studies reported that 19 to 65% of US and Canadian producers who raised dairy calves had treatment protocols (Schuler et al., 2017; Renaud et al., 2018; Okello et al., 2021). Hence, our findings align with previous research, but there may have been some bias in responses about having written protocols because of requirements under a mandatory national program for dairy producers in Canada (Dairy Farmers of Canada, 2020), although written treatment protocols are not specifically required. Moreover, only the presence of a written treatment protocol was noted in the current study, and details on the content of the protocol (e.g., frequency, criteria to start antimicrobial treatment) were not captured. We did not ask how often producers actually referred to or followed their treatment protocols. Studies have suggested that calf caretakers may not follow the treatment protocols and may make decisions based on their experience or deviate from the treatment protocols to adjust for specific cases with severe clinical signs (Webb et al., 2019; Cobo-Angel et al., 2021; Okello et al., 2021). It is unclear whether deviations from a written treatment protocol relate to more or less usage of antimicrobials and to outcomes for calf health, which should be studied in the future.

Respiratory and systemic signs, such as elevated breathing rate (76%), cough (62%), fever (65%), and eye or nasal discharge (41%), were commonly used to inform antimicrobial treatment decisions for respiratory disease; however, few respondents used lack of appetite (10%) and behavior (10%). Studies have found an association between calf behavior and bovine respiratory disease, where calves with bovine respiratory disease showed lethargy and isolation from other calves (Cramer et al., 2016; Knauer et al., 2017). Given that few producers considered appetite and behavioral signs in the current study, these criteria could be used as additional indicators when combined with other tools such as ultrasonography or clinical scoring systems to detect a respiratory case. We used indicators described by Love et al. (2014), which allocated points for each sign; however, another study suggested placing a higher weight on abnormal breathing to improve respiratory disease diagnosis (Buczinski et al., 2018). As different scoring systems are used to define a respiratory case between studies (Maier et al., 2019), a common standard should be developed and updated based on the best evidence possible. This could help to encourage knowledge transfer initiatives to better inform the criteria used by calf caretakers and reduce confusion by producers about signs that should be used in making decisions about AMU for respiratory disease.

The most common combination of case-specific indicators (fecal consistency, attitude, level of dehydration, fever) was used by 18% of respondents when treating calf diarrhea with antimicrobials. Most producers used antimicrobials by evaluating for fever, bloody stool (Gomez et al., 2017), or depressed attitude (Berge et al., 2009), or by using the veterinarian's judgments and response to previous treatment (McDougall et al., 2017). This finding corresponds with a study where producers were more likely to use antimicrobials in a severe diarrhea case presenting with combinations of abnormal fecal consistency and signs of systemic disease (Habing et al., 2016). Habing et al. (2016) reported that 13% of producers used antimicrobials for a mild diarrhea case (loose feces, normal temperature, good appetite), 37% used antimicrobials for a moderate case (watery feces, normal temperature, good appetite), and 67% used antimicrobials for a severe case (watery feces, normal temperature, depressed attitude and decreased appetite). The use of antimicrobials in mild to moderate cases suggests that dairy producers may be making less prudent decisions on AMU in calf diarrhea when assessing solely fecal consistency. In this study, 11% of producers who used antimicrobials for calf diarrhea applied only fecal consistency. Through targeted antimicrobial therapy using a combination of case-specific indicators, AMU can be reduced by more than 80% on some farms while not adversely affecting the mortality risk of calves (Gomez et al., 2017). Having a written treatment protocol with input from the herd veterinarian could aid in making decisions on AMU for targeted cases.

Producers with medium herd sizes were less likely to consider systemic signs when making decisions on AMU for calf diarrhea compared with smaller farms. A review that targeted farms worldwide found that larger farms were more conscious of AMU and AMR, but the reason for this finding remains unknown (Farrell et al., 2021). The higher likelihood of medium-sized herds making decisions not based on systemic sign may be partly explained by the lack of dedicated labor on these farms. Nearly half of producers perceive it difficult to spend enough time on good calf care because of the prioritization of cows over calves (Santman-Berends et al., 2014; Wilson et al., 2021), which could lead to a challenge for labor prioritization on medium-sized farms. However, morbidity risk and decisions about antimicrobial treatment in calves are complex and affected by multiple management practices, such as colostrum feeding and bedding materials (Al Mawly et al., 2015; Chamorro et al., 2017), which we did not measure. We encourage further research to investigate the relationship among the number of calves per caretaker and their time available per calf, how disease is detected, and treatment decisions made.

There are some limitations to consider when interpreting the results of this study. First, our sample was based on producers who agreed to be part of a multiyear study on AMR, so inclusion bias is possible. The farms included in the study did not represent all farms that are in the Canadian dairy industry and could have resulted in uneven representation of the industry; therefore, the results should be interpreted with caution. In addition, our data were collected as part of a larger study, and we were limited to a small number of questions. However, to our knowledge, this is the first study to explore case-specific indicators for calf disease treatment decisions made by Canadian dairy producers. Furthermore, these findings highlight opportunities for knowledge transfer to improve prudent AMU and generate hypotheses for more detailed study of treatment decision-making.

In conclusion, antimicrobials are commonly used to treat calf diseases on Canadian dairy farms. Many producers used combinations of indicators to treat respiratory disease and diarrhea in calves. In addition, few farms indicated they had written treatment protocols for calf diseases. Based on this research, there appears to be an opportunity to refine AMU for calf diarrhea and respiratory disease. Subsequent research should investigate the treatment decision-making process and validate interventions to implement best practices.
